# Effect of *Carum copticum* nano-essence against *Saprolegnia* and *Fusarium*, and the Use of Multiplex PCR Assay for the Detection of These Organisms in Rainbow trout *Oncorhynchus mykiss*

**DOI:** 10.22092/ari.2020.342066.1450

**Published:** 2021-06-01

**Authors:** P Tour SavadKouhi, H Ahari, A. A Anvar, B Jafari

**Affiliations:** 1 Department of Food Sciences and Technology, Science and Research Branch, Islamic Azad University, Tehran, Iran; 2 Department of Food Hygiene, Science and Research Branch, Islamic Azad University, Tehran, Iran; 3 Department of Biology, Science and Research Branch, Islamic Azad University, Tehran, Iran

**Keywords:** *Carum copticum*, *Fusarium*, Multiplex PCR, Nano-essence, *Oncorhynchus mykiss*, *Saprolegnia*

## Abstract

This study aimed to investigate the fungal species isolation and confirmation by the Multiplex PCR method in aquatic fish. Evaluation of the inhibitory effect of nano-essential oils of *Carum copticum* on isolated fungal species was also conducted in this study. The PCR results showed that 3 out of 5 samples were diagnosed with *Fusarium solani*, and two of them were positive for *Saprolegnia*. Moreover, in 0.1% of the females' nanoparticles, one peak appeared that showed a particle with an average diameter of 360 nm, and two nanoparticles showed a peak with a mean diameter of 242 nm. The results of minimum inhibitory concentrations (MIC) and minimum fungicidal concentrations (MFC) showed that 0.01% nano essential oil had 0.08 and 0.07 mg/ml MIC values against *Fusarium solani* and *Saprolegnia*, respectively. Gram/ml was on the growth of *Fusarium solani* species. The essential oils of female plants had an MIC of 0.07 in 0.1% essential oil and 0.03 mg/ml in 0.01% essential oil in *Saprolegnia*. Furthermore, in the case of 0.1% nano essential oil, the results showed the MIC values of 0.04 and 0.03 mg/ml against *Fusarium solani* and *Saprolegnia*, respectively. The MFC values of 0.1% nano essential oil were 0.1 and 0.07 mg/ml against *Fusarium solani* and *Saprolegnia*, respectively. It was not found on *Fusarium* and *Saprolegnia*. Overall, the results of this study using PCR for direct detection showed that 70% and 50% of the samples were *Fusarium solani* and *Saprolegnia* positive, respectively; therefore, the PCR was an efficient method for the detection of fungi. According to the results of nano-essential oil (0.1%) of females, this nano-essence had a strong inhibitory effect on *Fusarium solani* and *Saprolegnia*.

## 1. Introduction

Aquatic water moulds are potential sources of foodborne diseases that can be isolated from a wide range of aquatic animals. They are ubiquitous in water supplies and cause a lot of losses in the aquaculture industry across the world. *Saprolegnia* and *Fusarium* are present in freshwater ecosystems; moreover, they are regarded as one of the most important parasites fungi in aquatic animals that cause illnesses in fish, fish eggs, shrimp, and sea turtles (Raeisi et al., 2016). 

In the field of aquaculture, the numbers of antifungal agents and replacements are limited since Formalin and Malachite green are widely used in the prevention of fungal infections in fish cultivation and fish egg hatching. Furthermore, the continued use of antifungal agents can resist pathogens and increase genetic mutation as well as accumulation in the aquatic body, which is very difficult to reduce or eliminate from food or the food production chain. This also causes great risks to consumers and the environment. Therefore, it is necessary to manage the fungal infections, followed by the development of more convenient and less risky treatments in aquatic life and the aquaculture industry (Nagaraja et al., 2016).

Nowadays, there is a need for more environmentally safe treatment methods; accordingly, there have been more interest in scientists to pay attention to traditional medicine to overcome the pathogenic agents and problems of industrial medicine. Medicinal herbs are often used because of their potential with antimicrobial properties; in addition, they are not accumulated in the body and have no side effects (Weisany et al., 2019). 

Several researchers have reported that *Trachyspermum ammi L.* (A traditional plant containing essential oil) and their derived compounds exhibit significant antifungal activity which can have therapeutic potential against fungi, such as Aspergillus species, *Candida albicans*, *C. utilis*, and *Saccharomyces cerevisiae*. 

*T. ammi*, which is known as ajowan, is widely distributed and cultivated in various regions, such as Iran, Pakistan, Afghanistan, and India. Hooshangi et al. (2016) have demonstrated several medicinal herbs, such as *Carum copticum*, which has inhibitory effects on the growth of aquatic *Saprolegnia parasitica* fungi. Moreover, they suggested that natural products had the potential to be used as antifungal compounds in the aquaculture industry. Similalry, Salimian et al. (2017) in their study showed that natural products derived from *Myrtus communis L.* (medicinal plant) had antifungal effective compounds against *Saprolegnia* and *Fusarium* isolated from the rainbow trout eggs. The results of this study also revealed that medicinal herbs could be considered a potential candidate for designing antifungal agents for the treatment of fish fungal infections. 

To date, the PCR methods have been developed since they dramatically reduce the time and cost of analyzing different types of samples. Multiplex PCR is one of the known rapid and specific molecular methods with high accuracy and sensitivity for the detection of target DNA molecules in a complex mixture among researchers. Additionally, it has been used successfully to monitor fungal species, and it is considered an alternative to conventional microbiological methods in the detection of fungi. Ghiasi et al. (2014) reported that molecular methods were the best techniques for the identification of *Saprolegnia* species (spp.) in aquatic animals using the RAPD-PCR method.

Today, fungal infections in aquatic animals are considered an important threat to the world which should be given sufficient attention. To date, various fungal infections have been increased due to intensive aquaculture with stressful culture conditions. However, *Carum copticum* nano-essence has been utilized so far only for the detection of treatment of a few numbers of pathogenic fungi in aquatic animals, and PCR methods have been applied for the identification of fungal spp. (Sim et al., 2019). 

The present study aimed to assess the molecular identification and isolation of *Saprolegnia* and *Fusarium* of *Oncorhynchus mykiss* fish using the Multiplex PRC method; moreover, an evaluation was conducted to investigate the inhibitory effect of *Carum copticum* nano-essence against these two organisms. 

## 2. Material and Methods

### 2.1. Fungal Strains and Culture

Fungal strains were isolated from the infected tissue of the rainbow trout (*O. mykiss*) purchased from a farm in Tehran, Iran. The isolation of fungal spp. was carried out as follows: 

Totally, 20 μL of fungal resulting mycelium was inoculated in the center of a 90-mm Petri dish containing 25 mL of solidified potato dextrose agar (PDA; Difco, Franklin Lakes, NJ, USA) and incubated at 25°C for seven days. It should be mentioned that these isolates were maintained on PG-1 agar medium. 

### 2.2. DNA Extraction

Pure fungal culture was utilized for the DNA extraction using an UltraClean Microbial DNA isolation Kit (Mo Bio Laboratories, USA) according to the manufacturer's instructions. Afterward, the template DNA was extracted from the fungal mycelia collected from seven-day-old cultures incubated at a PDA medium at 25°C. To determine the concentration and purity of the extracted DNA samples, an Epoch™ microplate spectrophotometer was used by absorbance reading at A260/A280 ratio (BioTek, Winooski, VT, USA). Subsequently, the purified DNA was stored at -20°C until further use (Stakheev et al., 2011).

### 2.3. Primers and PCR Condition

For the identification of the strains, two specified primers were designed according to the Mitochondrial Cytochrome *B* gene presented in the ZEA biosynthesis cluster based on the published gene sequences. The primer sequences together with amplicon sizes are presented in Table 1. 

**Table 1:** Primer sequences and amplicon sizes used in this study

Primer	Nuleotic sequence	Target genes	size (bp)	Reference
f fuso1	5’-CTC TGT TAA TAA TGC AAC TC-3'	mitochondrial cytochrome b	330	(O'Donnell et al., 2010)
R fuso 2	5'-TGG TAC TAT AGC TGG AGG A-3'			
fusarium spp1	5’-AGT ATT CTG GCG GGC ATG CCT GT-3’		357	
fusarium spp2	5’-ACA AAT TAC AAC TCG GGC CCG AGA-3’			
ITS1	5'-TCCGTAGGTGAACCTGCGG-3'	ITS	700	(Liu et al., 2017)
ITS4	5'- TCCTCCGCTTATTGATATGC-3'			

 A Multiplex PCR assay containing total primers was performed using a T100™ Thermal Cycler (Bio-Rad, Hercules, CA, USA). In total, 20 μL of premixed PCR buffer (AccuPower™ PCR premix; Bioneer, Daejeon, Korea) was applied for this assay. Each PCR mixture containing 1-20 ng of genomic DNA as a template, 1.5mM MgCl2, 10mM Tris-HCl (pH 9.0), 30mM KCl, 250 μM of each dNTP, and 1-10 μM of forward and reverse primers along with 1 unit of Taq polymerase (Merck Millipore, Billerica, MA, USA) was used in this study. The Multiplex PCR approach was carried out as follows: 

Initial denaturation at 94°C for 4 min, followed by 35 cycles of the subsequent three steps of denaturation at 94°C for 1 min, annealing at 64°C for 30 sec, and extension at 72°C for 1min, as well as a final extension at 72°C for 10 min. Afterward, the PCR products were mixed with loading STAR buffer (Dyne Bio, Korea) and loaded on each column of the gel which was prepared from 3.0% agarose gel in Tris-acetate EDTA buffer (TAE; Bio-Rad). In the next stage, the electrophoresis was run at 100 Volts for 50 min while applying a 100-bp DNA as a marker. Finally, the DNA bands were visualized using a Gel Doc™ EZ imager (Bio-Rad) (Suanthie et al., 2009).

### 2.4. Preparation of the Plant Extract

Ajwain seeds were purchased from a local herbal store in Tehran, Iran, and dried in a warm air oven at 50°C to a persistent weight. Subsequently, all seeds were broken up into a fine powder using a kitchen blender. All powdered seeds which passed through an 80-mesh sieve were then taken for use. To prepare plant extracts, 20 g of crushed seeds were mixed with 200 ml of 85% ethanol and kept in a shaking water bath for 24 h at room temperature. The extract was then separated from the solid leftover using a filter paper (Whatman No. 1). The residual was re-extracted two times, and the extracts were pooled. A rotary vacuum evaporator (Laborota 4000, Heidolph, Germany) at 30°C was applied to remove the solvent. All extracts were kept at -80°C till further use (Mobaiyen et al., 2015).

### 2.5. Essential Oil Extraction

To obtain the total recovery of oil, 100 grams of each sample were mixed with 500 ml of distilled water using a Clevenger-type apparatus for 3 h. The preparation of the essential oil (EO) was repeated three times, and the extracted oils were dried up in the excess of sodium sulfate and weighed before putting them at 4°C until use (Chahal et al., 2017). 

### 2.6. Gas-chromatography-mass Spectrometry

An Agilent 6890 gas chromatograph-mass spectrometer (GC/MS) combined with an HP-5MS capillary column (30 m×0.25 mm) connected with an Agilent 5973 mass spectrometer (Agilent Technologies, Palo Alto, Canada) were utilized to analyze the extracted EOs. For the identification of the compounds, the recorded mass spectra were matched with the mass spectra in the data bank (Wiley 7N library). 

### 2.7. Preparation of the Nano-Encapsulated Essence

For nanogel production, 0.5 g chitosan was solved in 1% acetic acid (pH=3-3.5) and mixed with a magnetic stirrer to yield 0.5% chitosan solution. The solution was then sonicated for 20 min to be homogenized. After adding Carbodiimide myristic acid to the chitosan solution, 0.1 M sodium hydroxide was applied to adjust the pH in the range of 4.5-6.5. The obtained viscous gel was centrifuged three times before the absolute ethanol was added to remove any contamination. Finally, to get 5000 ppm of nano-essence, 25 µL essence was added to 5000 µL of nanogel and left in an ultra-sonicate device for 5 min (Hadian et al., 2016).

### 2.8. Assay of Nano Capsule with a Particle Size Analyzer

A Particle Size Analyzer was utilized to confirm the nano-essence sizes and prepare the samples. 

### 2.9. Antimicrobial Activity

Minimum inhibitory concentrations (MIC) and minimum fungicidal concentrations (MFC) were determined using the microdilution method in 96-well microplates as defined by Daouk et al. (1995) (Rodríguez-Tudela et al., 2003). The fresh overnight cultures of fungi were adjusted with sterile saline to have a concentration of 1.0×10^4^ CFU per well. The EOs were added into the sabouraud dextrose broth medium, and the 96-well microplates were incubated for 48 h at 37°C. 

The MIC was identified as the lowest concentration of EO preventing the visible growth of the test strain. However, after adding 40 µL of iodonitrotetrazolium violet 0.2 mg/ml, the ratio values of MIC/Minimum Bactericidal Concentration (MBC) for fungi were detected and incubated at 37°C for 30 min. The MBCs/MFCs were determined by serial sub cultivations of 10 µL into microtiter plates containing 100 µL of broth per well and further incubation for 24 h at 37°C. The lowest concentration with no visible growth was defined as the MFC, implying 99.5% killing of the original inoculum. Each test was conducted in triplicates; moreover, two antibiotics (Streptomycin and mycotic) (Fluconazole) were used as a positive control in both experiments (Tabassum Khan and Jameel, 2018).

### 2.10. Statistical Aanalysis

Analysis of variance, followed by Duncan's multiple range test (P<0.05) were performed in SPSS software (version 19; IBM SPSS Statistics, IBM Corporation, Armonk, NY, USA) to evaluate the ZEA levels detected in fungal cultures.

## 3. Results

**Figure 1:**
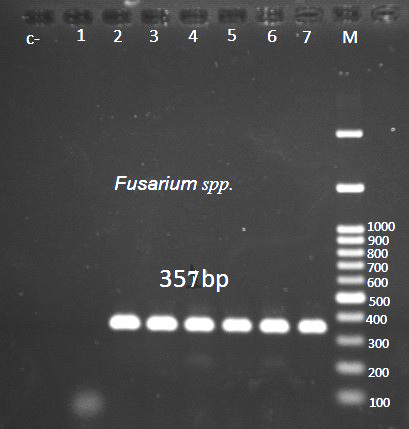
Result of electrophoresis to identify *Fusarium*

**Figure 2:**
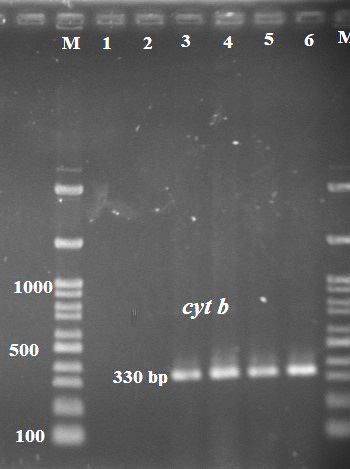
Result of electrophoresis to identify *Fusarium* spp.

**Figure 3:**
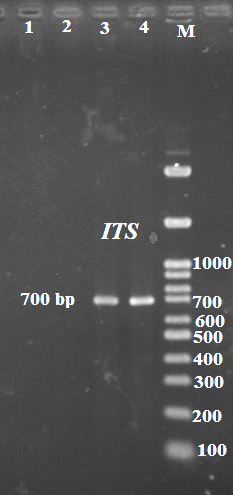
Result of electrophoresis to identify *Saprolegnia*

Mycological analysis of the samples revealed the incidence of *Fusarium* and *Saprolegnia* spp. Reference strains were confirmed through Multiplex PCR analysis using one forward primer and two reverse primers. Electrophoresis to identify *Fusarium* and its spices are shown in Figures 1 and Figure 2, respectively. Moreover, Figure 3 presents the electrophoresis results to identify *Saprolegnia*.

### 3.1. Chemical Composition of the Essential Oil

The GC-MS spectrometry analysis of the total essential oil of *Carum copticum* led to the identification and quantification of 11 chemical components. The major components were dominated by thymol (45%), p-cymene (25%), and g-terpinene (18%) in descending order. Table 2 presents the chemical compositions related to the *Carum copticum* essential oil. 

**Table 2:** Chemical compositions related to the *Carum copticum* essential oil

Number	Components	Concentrations (%)
1	Thymol	45%
2	𝑝-Cymene	25%
3	𝛾-Terpinen	18%
4	𝛽-Pinene	1.3%
5	𝛽-Phellandrene	0.7%
6	Sabinene	0.7%
7	𝛽-Myrcene	0.5%
8	𝛼-Terpinene	0.4%
9	Terpinene	0.3%
10	𝛼-Pinene	0.3%
11	𝛼-Nogen	0.3%

### 3.2. Evaluation of MIC and MFC

Antifungal activity of *Carum copticum* seeds nano-essence against the reference organisms is demonstrated in Table 3. The examination of the antifungal activities by the broth microdilution method exhibited that nano-essence had more activities against *Saprolegnia*, compared to *Fusarium*. The results showed that the MIC values of the essential oil ranged from 0.04 to 0.03 mg/ml against *Fusarium* and *Saprolegnia*, respectively. Moreover, the MFC values were ranging from 0.1 mg/ml against *Fusarium* to 0.07 mg/ml against *Saprolegnia*.

**Table 3:** Results of MIC and MFC against the *Fusarium* and *Saprolegnia*

Microorganism	MIC Percent of 0.01	MIC Percent of 0.1	MFC Percent of 0.01	MFC Percent of 0.1
Fusarium	0.08	0.04	0	0.1
Saprolegnia	0.07	0.03	0	0.07

## 4. Discussion

There have been many reports that pathogenic fungi are the most important acute and chronic fish infections. The number of medications is low, and drug resistance is increasing; accordingly, the tendency for natural alternative therapies has increased. Multiplex assays have been applied for the detection of many samples, including animal and human tissues for the presence of bacteria, parasites, viruses, and fungi. Niessen et al. (2004) reported that PCR methods were very useful for rapid and sensitive detection as well as identification and characterization of some genes in fungal species. 

In our study, PCR assay revealed the incidence of *Fusarium* and *Saprolegnia* spp. Similar to our study, Gevezova et al. (2017) demonstrated the power of m-PCR assay as molecular diagnostic method for bacterial pathogen detection in fish farms; moreover, their results were in line with the findings of other studies to develop an m-PCR assay for the detection of fish pathogens (Sim et al., 2019).

There have been reports of pathogenic fungi in adult fish species in the African continent. Our current result has similarities with the findings of the study performed by Florio et al. who revealed six fungal genera isolated from *Clarias gariepinus* eggs and broodstocks which *Fusarium* and *Saprolegnia* spp. were also part of them. Moreover, *Fusarium* and *Saprolegnia* spp. were isolated from ornamental fish Fantail, as well as diseased and apparently healthy fish samples. In addition, Shahbazian et al. (2010) isolated 17 species of fungi from the rainbow trout eggs.

Although most fungi are considered opportunistic pathogens, few of them are known to cause diseases, such as *Saprolegniasis*, which is caused by *Saprolegnia* fungi. In the current study, it was possible to identify the genus *Saprolegnia* from rainbow trout *O. mykiss*. It should be noted that this finding is consistent with the results of many other reports.

The obtained results of chemical constituents of the essential oil of *C. copticum* seed examined by the GC-MS analysis indicated 90.4% of the total amount of the essential oil that contains 11 components, including thymol (45.1%), p-cymene (25.8%), 𝛾-terpinene (18.2%), and 𝛽-pinene (1.3%). *C. copticum* grows in different areas of the world and contains different compound s. Similar to the findings of several studies, our results also confirmed that the main and major components of the Iranian *C. copticum* oil are thymol, 𝛾-terpinene, and p-cymene. (Boskabady et al., 2014).

Antifungal activity of the essential oil of *C. copticum* seeds is documented against toxigenic fungi species. According to the results, our study also revealed that *C. copticum* seed nano-essence possesses antifungal potency against *Fusarium* and *Saprolegnia* which is maybe due to the content present in *C. copticum* extract. *C. copticum* EO was the most active with MIC and MFC values ranging from 0.03 to 0.04 mg/ml and 0.1 to 0.07 mg/ml, respectively (Goudarzi et al., 2011). 

## 5. Conclusion

The present study has shown that the nano-essence of *T. ammi* possesses antifungal activity due to the presence of its organic compounds. Therefore, the development of rapid methods for the identification of pathogenic fungi in aquatic animals and the use of appropriate natural alternative therapies is evident in this regard. Such bioactive molecules could be utilized for the synthesis of antifungal agents.

## Authors' Contribution

Study concept and design: H. A. 

Acquisition of data: H. A.

Analysis and interpretation of data: H. A.

Drafting of the manuscript: H. A.

Critical revision of the manuscript for important intellectual content: H. A.

Statistical analysis: H. A.

Administrative, technical, and material support: H. A.

## Ethics

We hereby declare all ethical standards have been respected in preparation of the submitted article.

## Conflict of Interest

The authors declare that they have no conflict of interest.

## Grant Support

This study has no sponsorship.
